# Hemocompatibility-related Adverse Events Following HeartMate II Left Ventricular Assist Device Implantation between Japan and United States

**DOI:** 10.3390/medicina56030126

**Published:** 2020-03-13

**Authors:** Teruhiko Imamura, Minoru Ono, Koichiro Kinugawa, Norihide Fukushima, Akira Shiose, Yoshiro Matsui, Kenji Yamazaki, Yoshikatsu Saiki, Akihiko Usui, Hiroshi Niinami, Goro Matsumiya, Hirokuni Arai, Yoshiki Sawa, Nir Uriel

**Affiliations:** 1 Second Department of Medicine, University of Toyama, Toyama 930-0194, Japan; kinugawa-tky@umin.ac.jp; 2 Department of Medicine, University of Chicago Medical Center, IL 60637, USA; nuriel@medicine.bsd.uchicago.edu; 3 Department of Cardiac Surgery, University of Tokyo Hospital, Tokyo 113-8654, Japan; minoruono61@hotmail.com; 4 Department of Transplant Medicine, National Cerebral and Cardiovascular Center, Osaka 564-8565, Japan; nori@ncvc.go.jp; 5 Department of Cardiovascular Surgery, Kyushu University, Fukuoka 812-8582, Japan; shiose@heart.med.kyushu-u.ac.jp; 6 Department of Cardiovascular and Thoracic Surgery, Hokkaido University Graduate School of Medicine, Hokkaido 060-0808, Japan; ymatsui@med.hokudai.ac.jp; 7 Department of Cardiovascular Surgery, Tokyo Women’s Medical University, Tokyo 162-0054, Japan; yamaken964@gmail.com; 8 Department of Cardiovascular Surgery, Tohoku University, Miyagi 980-0812, Japan; yoshisaiki@med.tohoku.ac.jp; 9 Department of Cardiovascular Surgery, Nagoya University Hospital, Nagoya 464-8601, Japan; ausui@med.nagoya-u.ac.jp; 10 Department of Cardiovascular Surgery, Saitama Kokusai Medical Center, Saitama 350-1298, Japan; hniinami@saitama-med.ac.jp; 11 Department of Cardiovascular Surgery, Chiba University, Chiba 260-8677, Japan; matsumg@faculty.chiba-u.jp; 12 Department of Cardiovascular Surgery, Tokyo Medical and Dental University, Tokyo 113-8510, Japan; hiro.tsrg@tmd.ac.jp; 13 Department of Cardiovascular Surgery, Osaka University Graduate School of Medicine, Osaka 565-0871, Japan; sawa@surg1.med.osaka-u.ac.jp; 14 Division of Cardiology, Columbia University Medical Center, New York, NY 10019, USA

**Keywords:** heart failure, LVAD, HeartMate, bleeding, stroke

## Abstract

*Background:* Left ventricular assist device (LVAD) therapy has improved the clinical outcomes in advanced heart failure patients, however, this may differ between countries. We aimed to compare outcomes between Japanese and US LVAD cohorts. *Methods:* For 416 consecutive LVAD patients who received HeartMate II LVAD implantation and completed a one-year follow-up, age-matched Japanese patients (the Japanese registry for mechanically assisted circulatory support (J-MACS) group) and the US patients were compared for their clinical outcomes. *Results:* 154 J-MACS patients and 77 US patients were compared. Survival, free from hemocompatibility-related adverse events (HRAEs) in the J-MACS was statistically comparable with the US (75% vs. 63%, *p* = 0.79). J-MACS had more disabling strokes than the US (0.221 vs. 0.052/patient-year, *p* = 0.005), whereas there was less nonsurgical bleeding (0.045 vs. 0.117/patient-year, *p* = 0.024). The net hemocompatibility score was statistically comparable between the groups (1.54 vs. 1.19 points/patient, *p* = 0.99). Post-LVAD prothrombin time with international normalized ratio (INR) <1.5 (odds ratio 4.07) was a risk factor for HRAEs in J-MACS, whereas INR >3.0 (odds ratio 5.71) was a risk factor in the US (*p* < 0.05 for both). *Conclusion:* In the age-matched cohorts, the J-MACS group experienced more strokes, while the US group had more bleedings. “Tailor-made” therapeutic strategy might be required for each country, given the unique variation of HRAE incidence among each country.

## 1. Introduction

The improvement of continuous-flow left ventricular assist device (LVAD) technologies has resulted in a paradigm shift in the therapeutic strategy of patients with stage D heart failure (HF), from acute critical support to long-term performance-based goals [1]. However, LVAD technologies have also introduced various unique hemocompatibility-related adverse events (HRAEs) such as bleeding and thrombosis [2], consequently, HRAEs are major a cause of readmission following LVAD implantation [3]. 

Although there have been no comparison studies, incidences of individual HRAE may vary by country, for example, bleeding is known as one of the major causes of readmission in the US [3], but is rarely experienced in Japanese populations [4]. Such differences in outcomes may be driven by differences in obvious and nonobvious background characteristics, indications for LVAD implantation, and management protocols. 

A comparison of HRAEs between countries may lead to a better understanding of the mechanism underlying HRAEs, and thereby allow for the development of a “tailor-made” therapeutic strategy for each country. Accordingly, this study compared HRAEs between the Japanese registry for mechanically assisted circulatory support (J-MACS) and a large single-center registry in the US. 

## 2. Methods

### 2.1. Patient Selection

#### 2.1.1. US

Consecutive patients who underwent HeartMate II LVAD implantation at a large US academic center between April 2014 and January 2017 and completed a one-year follow-up were retrospectively included. All patients provided informed consent before the surgery. The study protocol was approved by the University of Chicago institutional review board on 12 July, 2016 (IRB16-0632). 

#### 2.1.2. J-MACS

Consecutive patients who received HeartMate II LVAD implantation at 30 Japanese institutions between April 2014 and January 2017 and completed a one-year follow-up in the J-MACS registry were included. All implantations were performed as a bridge to transplantation (BTT) [4], and all participants were ≤65 years old. Patients provided informed consent at each institution before the surgery. The use of the J-MACS registry was approved by the J-MACS committee on 15 May, 2018 (No. 180305). 

### 2.2. Follow-up Protocol

All patients received guideline-directed medical therapy including aspirin and warfarin with a target international normalized ratio (INR) of 2.0–3.0 in the US and 2.0–2.5 in Japan during the one-year follow-up period, unless HRAEs occurred [5]. Aspirin use was considered for all patients, unless they were considered to have a high risk of bleeding. Aspirin use and INR level one month following LVAD implantation was collected. LVAD speed data and mean arterial pressure at index discharge were obtained. During the one-year follow-up period, rates of mortality and HRAEs were examined. 

### 2.3. HRAE

Clinically adverse events attributable to LVAD-related bleeding or thrombosis were classified as an HRAE as detailed previously [6]: (1) Nonsurgical bleeding; gastrointestinal or other nonsurgical bleedings that occurred at >30 days following LVAD implantation. (2) Neurological events; stroke or other neurological events, which was diagnosed by neurological experts using image studies. (3) Thromboembolic events; pump thrombosis that was medically or surgically treated. The indication of medical treatment was lactate dehydrogenase increase over 2.5 holds over the normal range. Surgical device exchange was considered for those refractory to medications, with positive results of ramp test. All events were defined according to the INTERMACS definition. 

### 2.4. Hemocompatibility Score

A tiered hierarchal score (hemocompatibility score; HCS) was calculated for each patient by weighting each event considering its escalating clinical relevance, as detailed previously [6,7], to determine the aggregate net burden of HRAEs, instead of assessing individual types of event. The definitions of each tier and the corresponding weighted score are shown in Table A1. 

### 2.5. Statistical Analyses

Statistical analyses were performed with SPSS Statistics 22 (SPSS Inc., Armonk, IL, USA). Two-sided *p* values <0.05 were considered statistically significant. Continuous variables were described as median and quartiles and compared between groups using the Mann–Whitney U test. Categorical variables were compared between groups using Fisher’s exact test. 

Considering that age is a well-known critical determinant of HCS [7], a propensity score matching analysis was performed to select age-matched populations with a ratio of 1:2 of the J-MACS group and US group. A propensity score was calculated using logistic regression modeling and paired participants within 0.2 * standard deviation of all propensity scorings were selected. This age-matched population was a major cohort that we analyzed in this study. 

HRAE-free survival rates were assessed by using Kaplan–Meier analysis and compared between the groups by using the log-rank test. The distributions of HCS were compared between the groups by using Wilcoxon’s signed rank-sum test. Multivariable logistic regression analyses with the force-on method were performed on the variables with *p* < 0.10 in the univariate analyses to investigate significant factors associating with each endpoint. The primary outcome was freedom from any HRAEs. Secondary outcomes were event rates of each HRAE and HCS. 

## 3. Results

### 3.1. Baseline Characteristics of All Participants

A total of 419 HeartMate II LVAD patients (326 J-MACS and 93 US) were included (Table 1). J-MACS patients were younger (45 vs. 56 years old), had smaller body surface area (BSA; 1.65 vs. 2.13 m^2^), and had less ischemic etiology for HF (11% vs. 26%; *p* < 0.05 for all). Most of J-MACS patients were of Asian race (99%), and all received LVAD implantation as BTT. 

J-MACS patients received aspirin therapy at a higher rate than the US patients (89% vs. 80%) and their LVAD speed at index discharge was lower than the US patients (8600 vs. 9000 rpm) (*p* < 0.05 for both). 

### 3.2. Baseline Characteristics of Age-Matched Populations

Age-matched US patients (*n* = 77) and J-MACS patients (*n* = 154) were statistically selected and this was the main cohort in this study. Differences in other baseline characteristics were preserved except for the ischemic etiology and aspirin use (Table A2). 

### 3.3. Freedom From HRAEs Between the Age-Matched Groups

**Figure 1:**
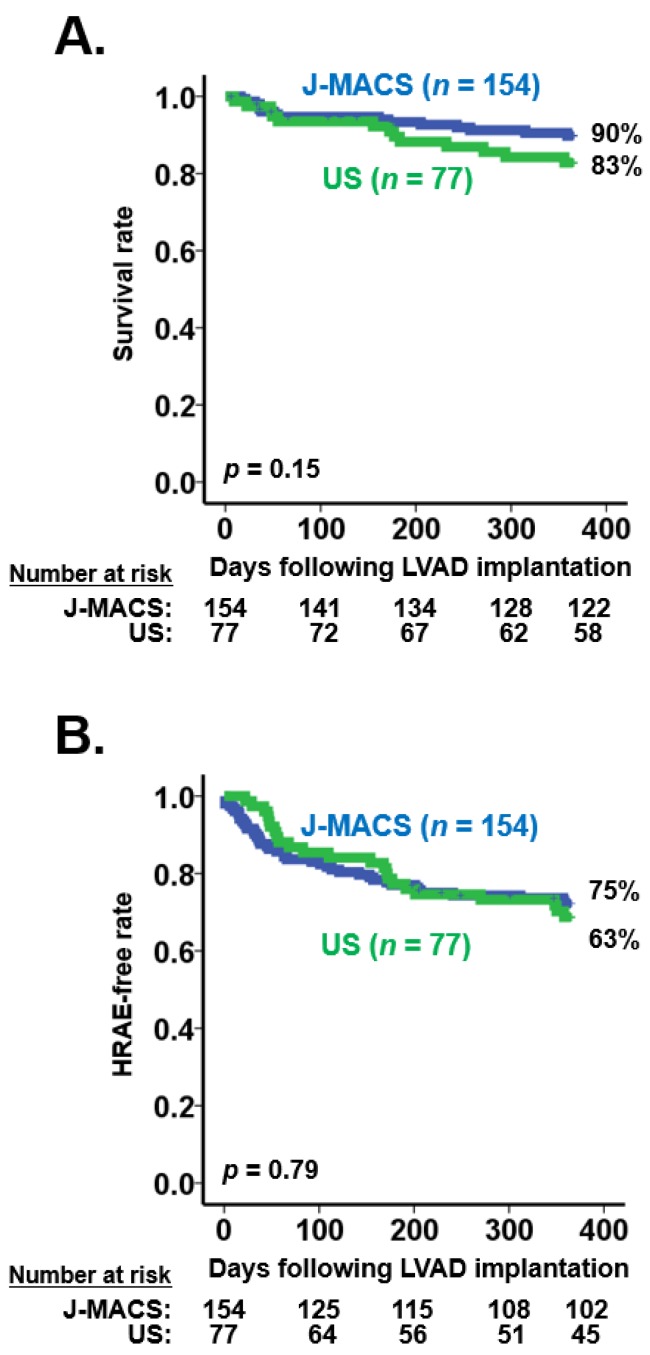
Survival rates (**A**) and HRAE-free rates (**B**) between J-MACS and US age-matched populations. HRAE, hemocompatibility-related adverse event; J-MACS, Japanese registry for mechanically assisted circulatory support.

There was no significant difference in overall survival (90% vs. 83%; Figure 1A) and HRAE-free rates (75% vs. 63%; Figure 1B) between the J-MACS and US groups (*p* < 0.05 for both). 

### 3.4. HCS Between the Age-Matched Groups

#### 3.4.1. Nonsurgical Bleeding (Tier I and Tier II)

Nine bleeding events in the US cohort and seven events in the J-MACS cohort occurred (Table 2). The bleeding event rate was 2.5-fold higher in the US than J-MACS groups (0.117 vs. 0.045 events/patient-year, *p* = 0.024). 

#### 3.4.2. Stroke (Tier II and Tier IIIB)

Disabling stroke rate in the J-MACS cohort was 4-fold higher than the US cohort (0.221 vs. 0.052 events/patient-year, *p* = 0.005) (Table 2). Four J-MACS patients experienced repeated disabling strokes, whereas nobody in the US cohort experienced multiple events. 

#### 3.4.3. Pump Thrombosis (Tier I and Tier IIIA)

For medically managed pump thrombosis, three events in the J-MACS cohort, and nine events in the US cohort occurred (Table 2). For surgically managed pump thrombosis, eight events in the J-MACS cohort, and nine events in the US cohort occurred. The incidence of medically managed pump thrombosis was comparable between the two groups (*p* = 0.53). The event rate of surgically managed pump thrombosis was statistically not different between the US group and the J-MACS group (0.104 vs. 0.058 events/patient-year, *p* = 0.21). 

#### 3.4.4. HCS (all Tiers)

**Figure 2:**
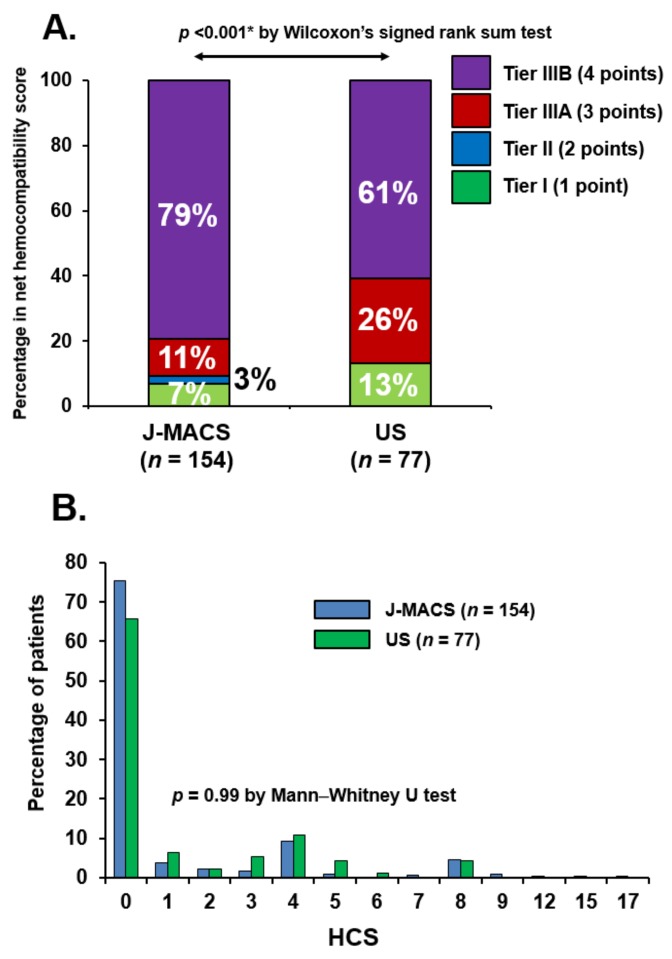
The proportional contribution of the Tiered events to the net HCS among age-matched populations (**A**) and distribution of net HCS in J-MACS and US among age-matched populations (**B**). The proportions of each Tier are different between the groups. The rate of Tier IIIB is higher in J-MACS, whereas the rates of Tier I and II are higher in the US (*p* < 0.005 * by Wilcoxon’s signed rank-sum test).

Percentages of each tier that contributed to the net HCS are shown in Figure 2A. The distributions of each tier differed between the groups (*p* < 0.001). Tier IIIB events were more prevalent in the J-MACS group (79% vs. 61%) due to a higher rate of disabling stroke. In contrast, Tier I (mainly nonsurgical bleeding, 13% vs. 7%) and Tier IIIA (surgically managed pump thrombosis, 26% vs. 11%) were more prevalent in the US group. However, net HCSs of both groups were comparably distributed (*p* = 0.99; Figure 2B).

### 3.5. Predictors of Death or Any HRAEs

For nonsurgical bleeding, INR >3.0 and non-Asian race were independent risk factors (*p* < 0.05 for both; Table A3). For stroke events, lower BSA, Asian race, and higher mean arterial pressure were significant risk factors (*p* < 0.05; Table A4), although we could not construct a significant multivariate model. 

**Figure A1:**
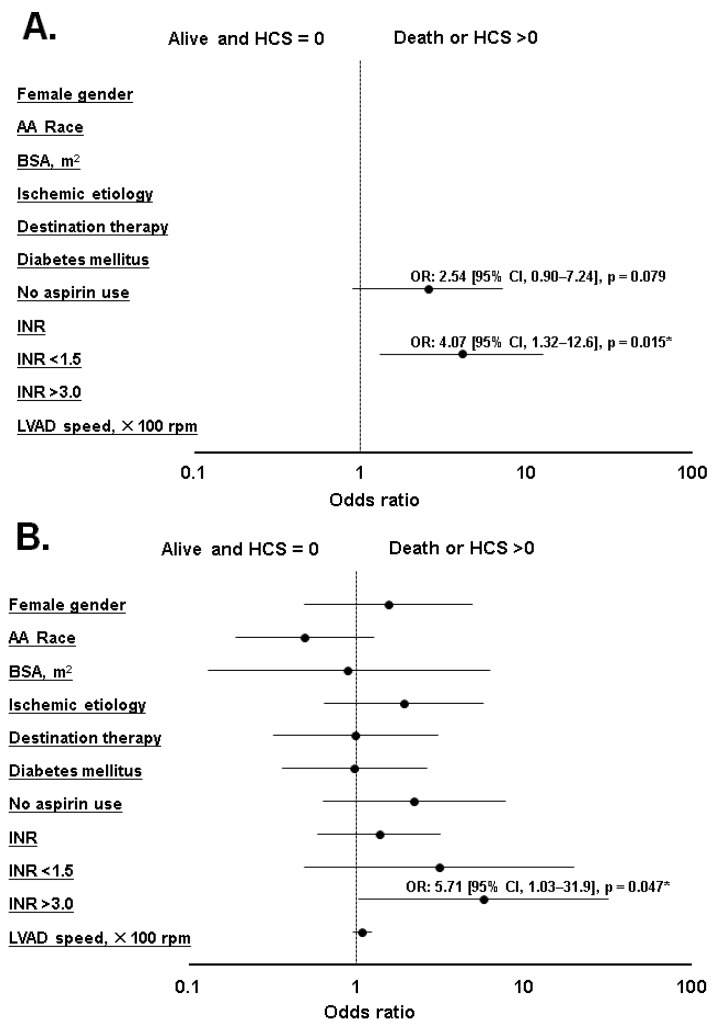
Predictors of death or any HRAEs among baseline characteristics in the J-MACS population in the multivariate analyses (**A**) and those in the US population in the univariate analyses (**B**). AA, African-American; BSA, body surface area; INR, prothrombin time with international normalized ratio; LVAD, left ventricular assist device; OR, odds ratio. * *p* < 0.05. Variables were assessed by using logistic regression analyses.

In the J-MACS group, no aspirin use, INR as a continuous variable, and INR <1.5 at one month were significant predictors of death or any HRAEs in the univariate analyses (*p* < 0.05 for all). Among them, INR <1.5 at one month was also significant in the multivariate model (odds ratio 4.07, 95% confidential interval 1.32–12.6, *p* = 0.015; Figure A1A). 

Among the US group, INR >3.0 at one month was a significant predictor of death or any HRAEs (odds ratio 5.71, 95% confidential interval 1.03–31.9, *p* = 0.047; Figure A1B). 

## 4. Discussion

We compared one-year survival and incidence of HRAEs following LVAD implantation between multiple Japanese institutions and a single US center among age-matched populations. Our main findings are as follows: (1) J-MACS patients were younger, and their LVAD speed at index discharge was lower. (2) Survival and freedom from HRAEs were comparable between the two groups when age was matched. (3) Higher rates of disabling stroke and lower rates of nonsurgical bleeding were observed in the J-MACS group compared with the US group. (4) INR <1.5 among the J-MACS group and INR >3.0 among the US group were risk factors for death or any HRAEs, respectively. 

### 4.1. The implication of the Comparison Between Japan and the US

The favorable results in the J-MACS registry, with the highest survival rate in the world (88.7% for two years) and low adverse event profile, might be the result of excellent patient care (both surgically and medically), optimal patient selection, and high post-discharge medical compliance [4]. Exploring the etiologies behind these favorable survival rates, post-LVAD implantation, would be beneficial to understand the driving forces behind these outcomes.

Age is an established risk factor of HRAEs. In the subanalysis of MOMENTUM 3 study, age was a significant predictor of death or any HRAEs during HeartMate 3 or HeartMate II support [7]. To focus more on other background differences between countries beyond age, we propensity-matched only age among the two groups in this study. Numerically lower survival in the US cohort might come from a higher prevalence of comorbidities and background severity, including destination therapy indication. 

### 4.2. Differences in Background Characteristics between Countries:

We identified several baseline characteristic differences between the J-MACS and US groups after age-matching. All J-MACS patients received LVAD implantation as BTT (destination therapy is not approved in Japan) [4]. Patients in the J-MACS registry had smaller BSA, and probably, as a result, LVAD speed was lower, although there is no study demonstrating the association between the body size and optimal device speed [8].

### 4.3. Less Bleeding in J-MACS

Our team recently demonstrated the key role of angiogenesis and inflammatory-related factors such as tumor necrotic factor-α and angiopoietin-2 in the formation of arteriovenous malformation, which contributes to gastrointestinal bleeding during LVAD support [9,10]. The lower rate of bleeding among the J-MACS population may be associated with multiple factors. One such factor might be genetic differences between the Caucasian and Asian ethnicities. Asian patients may have less activity in the angiogenesis-related signal cascade. We do not have direct evidence supporting this hypothesis, but one study reported that black (odds ratio 1.95) and Hispanic races (odds ratio 1.71) independently associated with significant blood loss in 4159 patients with documented colonic angiodysplasia [11]. Larger demographic studies within the field of mechanical circulatory support are sorely needed to better understand the true association of patient ethnicity and device-related adverse events.

Some data suggest that differences in diet may be associated with risks of bleeding. Japanese diets are generally rich in fish oil that contains unsaturated fatty acids, whereas this is less common in diets found in the US [12]. Our team recently demonstrated that omega-3, one of the unsaturated fatty acids, had a protective effect of gastrointestinal bleeding in LVAD patients. The mechanism likely relates to the suppression of angiopoietin-2 that is located upstream of the angiogenetic signal cascade [13]. However, we could not obtain any dietary data in this study. The investigation of inflammation and angiogenesis activities among Asian patients living in the US with a fish-less diet might clarify which factor (i.e., race vs. food) has more impact on bleeding.

In addition to the non-Asian race, INR >3.0 was another risk factor of bleeding (Table A3). Higher INR goal in the US might be another reason for higher bleeding incidence in the US. 

### 4.4. More Strokes in J-MACS

Mechanisms underpinning the higher stroke rate in the J-MACS are likely multifactorial. Multiple strokes were observed in the J-MACS group with aggressive life-prolonging strategies even after disabling strokes potentially capturing repetitive strokes after the initial event. Racial differences may also have a significant impact on the stroke rate. In the general population, it is well known that the stroke rate is higher in Japanese people compared with populations in Europe and the US [14,15]. Nevertheless, longitudinal data over a spectrum of populations are needed to better elucidate the association of race and incidence of stroke.

In addition to the Asian race, higher mean arterial pressure and smaller body surface area were other risk factors of stroke (Table A4). These might result in the development of stroke, particularly in the Japanese cohort. Of note, lower INR and LVAD speed were not significant risk factors of stroke. 

Although LVAD speed was lower in the J-MACS cohort, the incidence of pump thrombosis was numerically lower. We do not have any supporting data, but such a lower device speed might be sufficient for those with small physics like Japanese. The association between body size and optimal LVAD speed is a future concern. 

### 4.5. Optimal Therapeutic Strategy for Each Country

In this study, we used HCS to assess overall HRAE in addition to assessing individual HRAE, because we should find a balanced therapeutic strategy to prevent both bleeding and thrombosis equally during LVAD support. Given our findings, a lower INR was a risk factor of any HRAEs in the Japanese cohort, whereas a higher INR was a risk factor in the US cohort. Further longitudinal analyses are warranted to construct optimal INR ranges in each country. 

## 5. Limitations

J-MACS data were collected from 30 widely-distributed Japanese institutions, whereas US data were obtained from a single center. US data may not necessarily represent the overall US cohort, and a multi-center registry such as IMACS would be ideal. As a limitation of propensity score-matching analyses, statistically selected background-matched populations may not necessarily represent the original study population. Some comparisons did not reach statistical significance, but it does not mean similarity. A longer observational period might give us statistical significance. We focused on the HRAE in this study, and other comorbidities, including driveline infection, are the next targets to be studied. We should pay attention to interpreting our findings. A difference in the clinical outcome would dominantly come from various background differences, including comorbidities, management differences, and race, instead of a country difference itself. 

## 6. Conclusions

Japanese patients had more strokes, whereas US patients had more bleeding events. Construction of “tailor-made” therapeutic strategy that considers the unique difference in each country would be required. 

## Figures and Tables

**Table 1:** Baseline characteristics of all participants.

	J-MACS (*n* = 326)	US (*n* = 93)	*p* Value
Demographics			
Age, years	45 (35, 54)	56 (49, 67)	<0.001 *
Gender, male	250 (77%)	72 (77%)	0.50
Race			
White	0 (0%)	45 (48%)	<0.001 *
Black	0 (0%)	44 (47%)	<0.001 *
Asian	325 (99%)	1 (1%)	<0.001 *
Others	1 (1%)	3 (3%)	0.011 *
INTERMACS profile			0.55
Profile 1	33 (10%)	10 (11%)	
Profile 2	98 (30%)	26 (28%)	
Profile 3	182 (56%)	50 (54%)	
Profile ≥4	13 (4%)	7 (7%)	
Body surface area, m^2^	1.65 (1.54, 1.75)	2.13 (1.99, 2.32)	<0.001 *
Ischemic etiology	35 (11%)	24 (26%)	<0.001 *
Destination therapy	0 (0%)	73 (78%)	<0.001 *
Bridge to transplant	326 (100%)	20 (22%)	<0.001 *
Diabetes mellitus	64 (20%)	29 (31%)	0.055
Peripheral artery disease	2 (1%)	3 (3%)	0.11
Atrial fibrillation	48 (15%)	19 (20%)	0.19
History of strokes	46 (14%)	17 (18%)	0.39
Chronic obstructive pulmonary disease	4 (1%)	19 (20%)	<0.001 *
Medications and LVAD speed			
Aspirin use at one month (*n* = 409)	290 (89%)	74 (80%)	0.020 *
INR at one month (*n* = 405)	2.3 (2.0, 2.6)	2.3 (1.8, 2.6)	0.45
LVAD speed at discharge, rpm (*n* = 378)	8600 (8400, 8800)	9000 (8800, 9400)	<0.001 *
Mean arterial pressure at discharge, mmHg (*n* = 384)	74 (68, 82)	72 (66, 80)	0.048 *

INR, prothrombin time with international normalized ratio; LVAD, left ventricular assist device. * *p* < 0.05. Continuous variables were compared by using the Mann–Whitney U test; categorical variables were compared by using Fisher’s exact test.

**Table 2:** The number and rate of events contributing to each tier of net hemocompatibility score.

	J-MACS (*n* = 154)	US (*n* = 77)	*p* Value
Tier I			
Nonsurgical bleeding (≤2 events)	7 (0.045/patient-year)	9 (0.117/patient-year)	0.024 *
Medically managed pump thrombosis	9 (0.058/patient-year)	3 (0.039/patient-year)	0.53
Tier II			
Nonsurgical bleeding (>2 events)	0	0	1.0
Nondisabling stroke	2 (0.013/patient-year)	0	0.32
Tier IIIA			
Surgically managed pump thrombosis	9 (0.058/patient-year)	8 (0.104/patient-year)	0.21
Tier IIIB			
Disabling stroke	34 (0.221/patient-year)	4 (0.052/patient-year)	0.005 *
HC-related or inconclusive death	13 (0.084/patient-year)	10 (0.130/patient-year)	0.28

HC, hemocompatibility. * *p* < 0.05. Variables were compared by using the Mann–Whitney U test.

## Appendix A

**Table A1:** Calculations of hemocompatibility score.

Intensity	Clinical components	Score
Tier I: mild	≤2 gastrointestinal or other nonsurgical bleeding episodes	1 point each
	Suspected pump thrombosis (medically treated)	
Tier II: moderate	>2 gastrointestinal or other nonsurgical bleeding episodes	2 points each
	Nondisabling stroke	
Tier III		
IIIA: moderate to severe	Pump malfunction attributable to pump thrombosis leading to reoperation for removal or replacement	3 points each
IIIB: severe	Disabling stroke	4 points each
	Death attributable to a hemocompatibility etiology or inconclusive	

**Table A2:** Baseline characteristics in age-mated J-MACS and US populations.

	J-MACS (*n* = 154)	US (*n* = 77)	*p* value
Demographics			
Age, years	53 (48, 59)	53 (48, 60)	0.75
Gender, male	112 (73%)	59 (77%)	0.32
Race			
White	0 (0%)	30 (39%)	<0.001 ^†^
Black	0 (0%)	43 (56%)	<0.001 ^†^
Asian	153 (99%)	1 (1%)	<0.001 ^†^
Others	1 (1%)	3 (3%)	0.11
INTERMACS profile			0.77
Profile 1	15 (9%)	9 (12%)	
Profile 2	45 (29%)	20 (26%)	
Profile 3	88 (57%)	43 (56%)	
Profile ≥4	6 (4%)	5 (6%)	
Body surface area, m^2^	1.63 (1.52, 1.73)	2.14 (1.99, 2.33)	<0.001 *
Ischemic etiology	28 (18%)	17 (22%)	0.30
Destination therapy	0 (0%)	60 (78%)	<0.001 ^†^
Diabetes mellitus	42 (27%)	26 (34%)	0.48
Peripheral artery disease	1 (1%)	2 (3%)	0.37
Atrial fibrillation	26 (17%)	15 (19%)	0.63
History of strokes	25 (16%)	15 (19%)	0.54
Chronic obstructive pulmonary disease	3 (2%)	17 (22%)	<0.001 ^†^
Medications and LVAD speed			
Aspirin use at one month (*n* = 225)	131 (85%)	64 (83%)	0.28
INR at one month (*n*= 224)	2.3 (1.9, 2.6)	2.3 (1.8, 2.6)	0.91
LVAD speed at discharge, rpm (*n* = 205)	8600 (8400, 8800)	9000 (8980, 9400)	<0.001 *
Mean arterial pressure at discharge, mmHg (*n* = 201)	74 (69, 82)	72 (68, 80)	0.046 *

INR, prothrombin time with international normalized ratio; LVAD, left ventricular assist device. *p* < 0.05 * by Mann–Whitney U test, *p* < 0.05 ^†^ by Fisher’s exact test.

**Table A3:** Risk factors for any nonsurgical bleeding.

	Univariate Analysis		Multivariate Analysis	
	Odds Ratio (95% CI)	*p* Value	Odds Ratio (95% CI)	*p* value
INR > 3.0	5.11 (1.59–16.4)	0.006 *	5.53 (1.67–18.4)	0.005 *
No Asian race	2.78 (0.94–7.75)	0.051	2.92 (1.01–8.40)	0.048 *

* *p* < 0.05. Variables were analyzed by using logistic regression analysis. INR, prothrombin time with international normalized ratio.

**Table A4:** Risk actors for any stroke.

	Odds Ratio (95% CI)	*p* value
Body surface area, m^2^	0.11 (0.23–0.54)	0.006 *
Asian race	5.97 (1.76–20.2)	0.004 *
Mean arterial pressure, mmHg	1.05 (1.03-1.43)	0.010 *

* *p* < 0.05. Variables were analyzed by using logistic regression analysis.
